# Tumor-derived exosomes induce PD1^+^ macrophage population in human gastric cancer that promotes disease progression

**DOI:** 10.1038/s41389-018-0049-3

**Published:** 2018-05-25

**Authors:** Furong Wang, Bin Li, Yucai Wei, Yang Zhao, Li Wang, Peng Zhang, Jinwei Yang, Wenting He, Hao Chen, Zuoyi Jiao, Yumin Li

**Affiliations:** 1 Department of Pathology, Lanzhou University Second Hospital, Lanzhou University Second Clinical Medical College, Lanzhou, China; 2 Gansu Provincial Key Laboratory of Digestive System Tumors, Lanzhou University Second Hospital, Lanzhou University Second Clinical Medical College, Lanzhou, China; 3 School of Life Sciences, Lanzhou University, Lanzhou, 730000 China; 4 Department of Thoracic Surgery, Lanzhou University Second Hospital, Lanzhou University Second Clinical Medical College, Lanzhou, China; 5 School of Basic Medical Science, Lanzhou University, Lanzhou, 730000 China; 6 Department of General Surgery, Lanzhou University Second Hospital, Lanzhou University Second Clinical Medical College, Lanzhou, China

## Abstract

Macrophages constitute a major component of tumor-infiltrating immune cells. M2 macrophages have been reported to promote tumor progression through promoting tumor angiogenesis and metastasis and regulating T-cell function. Here, we identified a protumorigenic subset of macrophages that constitutively expressed programmed cell death 1 (PD1) and accumulated in advanced-stage gastric cancer (GC). These PD1^+^ tumor-associated macrophages (TAMs) exhibited an M2-like surface profile, with a significant increase in the expression of CD206, IL-10, and CCL1, and a clear decrease in the expression of MHC class II, CD64, and IL-12 and the ability to phagocytose ovalbumin. Moreover, PD1^+^ TAMs can suppress CD8^+^ T-cell function and this immunosuppressive activity can effectively be enhanced upon triggering PD1 signal. GC-derived exosomes effectively educated monocytes to differentiate into PD1^+^ TAMs with M2 phenotypic and functional characteristics. Together, our results are the first to show that GC-derived exosomes can effectively induce PD1^+^ TAM generation, and these cells can produce a large number of IL-10, impair CD8^+^ T-cell function, and thereby create conditions that promote GC progression. Thus, methods in which immunotherapy is combined with targeting PD1^+^ TAMs and tumor-derived exosomes should be used to restore immune function in GC patients.

## Introduction

Gastric cancer (GC), which is the fourth most common malignant tumor, is the third leading cause of cancer-related death in the world^1^. Helicobacter pylori (HP) infection is the main risk factor for the progression of chronic gastritis^2^ which is mainly composed of extracellular matrix, activated cancer-associated fibroblasts, and immune cells, such as myeloid-derived suppressor cells (MDSCs), tumor-associated macrophages (TAMs), regulatory T cells (Treg), and regulatory B cells (Breg), which accelerates GC progression. However, the key roles and underlying mechanisms of these immune cells are still understood.

Macrophages (Mφs) are a major component of tumor-infiltrating immune cells^3,4^, and associate with poor prognosis in tumor^5^. Recent studies have confirmed that TAMs can accelerate tumor angiogenesis^6^, metastasis^7^, and influence the T-cell activation and differentiation via the cytokine secretion^8,9^. However, TAMs have been confirmed to possess phenotypic plasticity which can be divided into the polarized states of M1 and M2^10^. The PD1 is one of the best-studied and most clinically successful immune-checkpoint drug targets^11^, but its function is well confirmed to regulate T-cell function. However, macrophages have previously been found to increase the PD1 expression during microbial infection^12–14^, and recent study has confirmed that TAMs also express PD1 and these PD1^+^ TAMs exhibit M2 phenotypic characteristics which favor tumor progression by inhibiting antitumor immunity^15^. Thus, we wondered whether GC-derived macrophages might also express PD1, and if so, how PD1^+^ TAMs are induced.

Exosomes are the small intraluminal vesicles with the diameter range from 30 to 200 nm. Exosomes have been reported to mediate local and systemic cell-to-cell communication through the delivery of information, such as microRNAs, mRNAs, cDNA, and proteins^16–19^. Tumor-derived exosomes have been reported to accelerate primary tumor growth^20,21^, and accelerate tumorigenesis^22^, tumor invasion, and metastases^23–25^ through the transfer of DNA, RNA, and proteins. Because of these observations, we investigate whether tumor-derived exosomes are involved in the PD1^+^ TAM production in GC progression.

## Materials and methods

### Human mononuclear cells separation

GC tissue was cut into small pieces with a size of about 0.1 cm and digested in RPMI 1640 containing 0.01% DNase I (Roche, Basel, Switzerland), 0.08% type IV collagenase (Sigma-Aldrich, St. Louis, Missouri, USA), and 15% fetal bovine serum (FBS) (HyClone, Ogan, Utah, USA). The digestion cells were filtered via a 150-μm filter, and then the mononuclear cells (MCs) were separated by density-gradient centrifugation. The MCs were collected and resuspended in PBS containing 1% heat-inactivated FBS for flow cytometry analysis. To analyze the function of PD1^+^ macrophages, PD1^+^ macrophages were purified from MCs using flow cytometry.

### Flow cytometry

The antibodies (Abs) were BV421, PE-, APC-, or FITC-conjugated mouse anti-human Abs: CD45, CD8 (#344704/344722), CD4 (#300508/300514), HLA-DR (#307606), CD206 (#321110), PD1 (#329906), CD11b (#101212), CD64 (#305008), IFNγ (#502506/502508), and perforin (#308112) from BioLegend (San Diego, CA, USA). The culture cells or MCs were collected and resuspended in PBS supplemented with 0.1% bovine serum albumin (BSA), and stained and labeled with either specific or isotype Abs at 4 °C for 30 min. Then, the cells were washed twice and fixed with 1% paraformaldehyde solution. Flow cytometry analysis was performed using FACScan and Flow Jo software. To analyze the intracellular expression of CD8^+^ T cells, 10 mg/ml PMA and brefeldin A (Sigma-Aldrich) was added at the last 4 h. The CD8^+^ T cells were collected, washed twice, and labeled with CD8 Ab at 4 °C for 30 min. Then, the cells were washed twice, fixed, and permeabilized with fixation/permeabilization solution (eBioSciences, San Diego, CA, USA) at room temperature for 15 min. Finally, the cells were washed once and labeled with perforin and IFN-γAbs at room temperature for 20 min.

### Enzyme-linked immunosorbent assay

Human IL-10, IL-12p70, and CCL1 concentrations of the culture media from macrophages were analyzed using Enzyme-linked immunosorbent assay (ELISA) kits following the manufacturers’ instructions (R&D Systems for CCL1; eBioscience for IL-12p70 and IL-10).

### Real-time polymerase chain reaction

Total RNA was extracted and determined using an SYBR Green real-time PCR kit. PCR products were determined by electrophoresis on 2% agarose gel and photographed after ethidium bromide staining.

### Immunofluorescence

Paraffin-fixed GC tissues were cut into 5-μm sections and carried out using a two-step protocol. In total, 5-μm sections were stained with the Abs: mouse anti-human CD68 (Dako), and rabbit anti-human PD1 (R&D Systems) at 4 °C for 16 h, followed by fluorescently labeled secondary Abs at 37 °C for 30 min.

### T-cell proliferation and cytokine production assays

To analyze the functions of PD1^+^ macrophages, 5,000,000 CD8^+^ T cells were cultured in 10 ml of PBS supplemented with 25 μM carboxyfluorescein succinimidyl ester (CFSE) (Invitrogen, Carlsbad, CA, USA) at 37 °C for 5 min, washed twice, and seeded at 100,000 cells in a 96-well round plate with 200 μl of RPMI 1640 medium supplemented with 10% FBS. PD1^+^ macrophages were obtained from tumor tissue using flow cytometry and added to CD8^+^ T-cell culture system at ratios of 1:1. Then, the CD8^+^ T cells were stimulated with 1 μl of anti-CD3 and 2 μl of anti-CD28 beads for 3 days. Finally, the proliferation and perforin and IFN-γ expression in CD8 T cells were determined using flow cytometry. In some experiments, PD1^+^ macrophages were pretreated with PD1 functional Ab (goat human-specific AF1086, R&D Systems) and total goat IgGs (R&D Systems) for 24 h, washed twice, and added to CD8^+^ T-cell culture system at ratios of 1:1.

### Endocytosis assay of macrophages

To evaluate the phagocytic ability, PD1^+^ macrophages were incubated for 30 min at 37 °C, with FITC-conjugated ovalbumin (OVA; 100 mg/mL; Sigma-Aldrich) in RPMI 1640 containing 10% FBS. Then, the cells were washed twice with PBS containing 0.5% BSA and 0.1% NaN_3_ and resuspended in PBS containing 0.1% NaN_3_ and 0.5% BSA for immediate evaluation by flow cytometry.

### GC exosome isolation and monocyte culture

Human gastric-cancer cell lines such as SGC7901 and BGC823, were purchased from the American Type Culture Collection (Rockville, MD, USA). Culture media from SGC7901 and BGC823 which were cultured with exosome-depleted serum were collected on day 3, and centrifuged at 1500 rpm for 10 min. Supernatants were centrifuged at 2500 rpm for 20 min, and then passed through 0.22-µm filters. Exosomes were obtained by mixing with ExoQuick-TC (System Biosciences) following the manufacturers’ instructions. Exosomes were resuspended in PBS, and total protein of exosomes was quantified by BCA assay. Isolated exosomes (50 µg) were added into the monocytes culture system for 3 days.

### Statistical analysis

The differences between groups were analyzed by Bonferroni post test. Correlations between groups were determined by Pearson’s correlation analysis as appropriate. A value of *p* < 0.05 was considered significant.

## Results

### PD1^+^ macrophages are enriched in gastric-cancer tissues and associated with poor patient survival

**Fig. 1:**
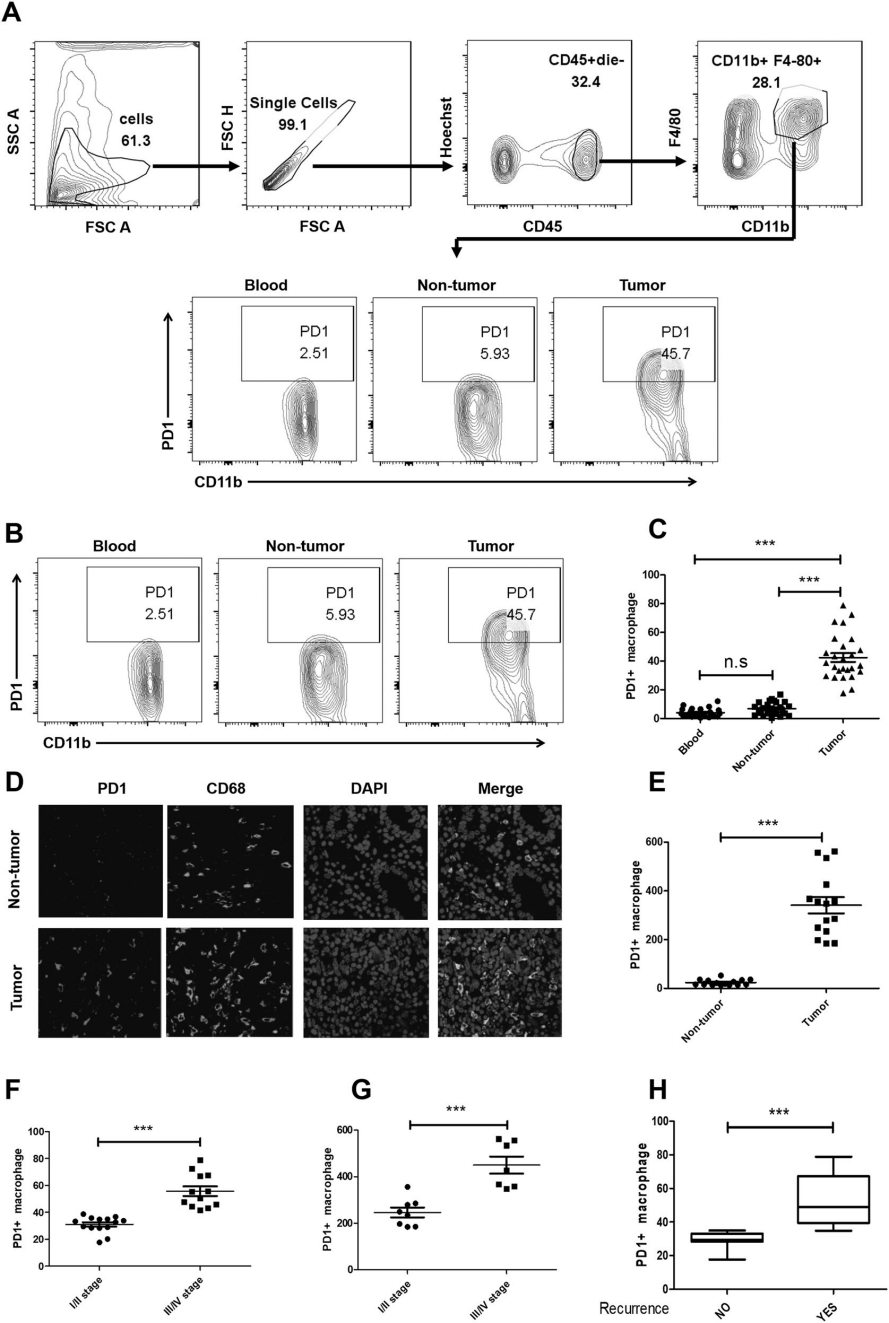
PD1 expression on GC-infiltrating macrophages and its clinical significance. **a** Flow cytometry gating strategy for GC-infiltrating macrophages. Debris and doublets were removed, and then TAMs were assessed as Hoechst−CD45+CD11b+F4/80+. **b**, **c** Analysis of PD1 expression on macrophages from the samples as follows: paired blood, non-tumoral gastric tissue, and tumor tissue from 26 patients. Representative FACS plots of macrophage PD1 expression are shown in (**b**). Frequencies of PD1^+^ macrophages relative to total macrophage frequencies are shown in (**d**). **d**, **e** Immunofluorescence analysis of the PD1 expression levels (red); and the CD68 expression levels (green) in GC tissue from 15 patients. Representative micrographs are shown in **d**. The density of tumor-infiltrating PD1^+^ macrophages is shown in (**e**). Associations of tumor PD1^+^ macrophages with GC patients’ TNM stage are shown in (**f**, **g**). **f** The proportions of PD1^+^ macrophages were determined by flow cytometry. **g** The number of PD1^+^ macrophages was determined by immunofluorescence staining. **h** Association of tumor PD1^+^ macrophages with early recurrence. Using 1 year as the cutoff, 20 GC were divided into two groups: no recurrence (*n* = 12) and recurrence (*n* = 8). *P < 0.01, **P < 0.001 (Student's *t* test)

To evaluate the PD1 expression of TAMs in an immunocompetent syngeneic setting, we first analyzed its expression pattern in GC tissues. We used flow cytometry to study the PD1 expression of macrophages in 26 GC specimens (paired blood, non-tumor tissue, and intra-tumor tissue; Table 1), and found that intratumoral macrophages expressed a significantly higher level of PD1 than those expressed on non-tumor tissue macrophages, whereas peripheral macrophages expressed little PD1 (Fig. 1a–c). Furthermore, we used immunofluorescence staining to study the expression of PD1 on macrophages in 15 GC specimens (paired non-tumor tissue, and intra-tumor tissue; Table 2). The results also showed that PD1^+^ macrophages were accumulated at GC tissue (Fig. 1d, e).

**Table 1:** Clinicopathological characteristics of gastric-cancer patients (*n* = 26)

**Variables**	**No. and (%)**
No. of patients	20
Age (median; range), years	59; 21–72
Gender (male/female)	17/9 (65.0/35.0)
Tumor size, cm (≤4/>4)	10/16 (40.0/60.0)
Tumor location (higher/middle/lower/other)	10/4/8/4 (40.0/15.0/30.0/15.0)
TNM stage (IA/IB/II/IIIA/IIIB/IV)	1/4/9/6/3/3 (5.0/15.0/35.0/25.0/10.0/10.0)
Gastric cancer subtype (common types/special type)	20/6 (77/23)

**Table 2:** Clinicopathological characteristics of gastric-cancer patients (*n* = 15)

**Variables**	**No. and (%)**
No. of patients	15
Age (median; range), years	56; 38–76
Gender (male/female)	9/6 (60.0/40.0)
Tumor size, cm (≤4/>4)	7/8 (46.7/53.3)
Tumor location (higher/middle/lower/other)	6/3/3/3 (40.0/20.0/20.0/20.0)
TNM stage (IA/IB/II/IIIA/IIIB/IV)	1/2/5/4/2/1 (6.7/13.3/33.3/26.7/13.3/6.7)
Gastric cancer subtype (common types/special type)	13/2 (86.7/13.3)

Next, we assessed the clinical relevance of intra-tumorous PD1^+^ macrophages in GC patients (Tables 1 and 2), and found that the proportions of PD1^+^ macrophages determined by flow cytometry were significantly associated with disease progression in 26 GC patients (Fig. 1f, Table 1), and that the number of PD1^+^ macrophages determined by immunofluorescence staining was also significantly associated with disease progression in 15 GC patients (Fig. 1g, Table 2). Importantly, using 24 months as the cutoff, we found that patients with higher proportions of PD1^+^ macrophages in tumor showed early recurrence of GC (Fig. 1h). Thus, these results show that PD1^+^ macrophages selectively accumulated at the tumor that associate with disease progression and early recurrence in GC patients.

### Phenotypic and functional characteristics of PD1^+^ macrophages in GC patients

**Fig. 2:**
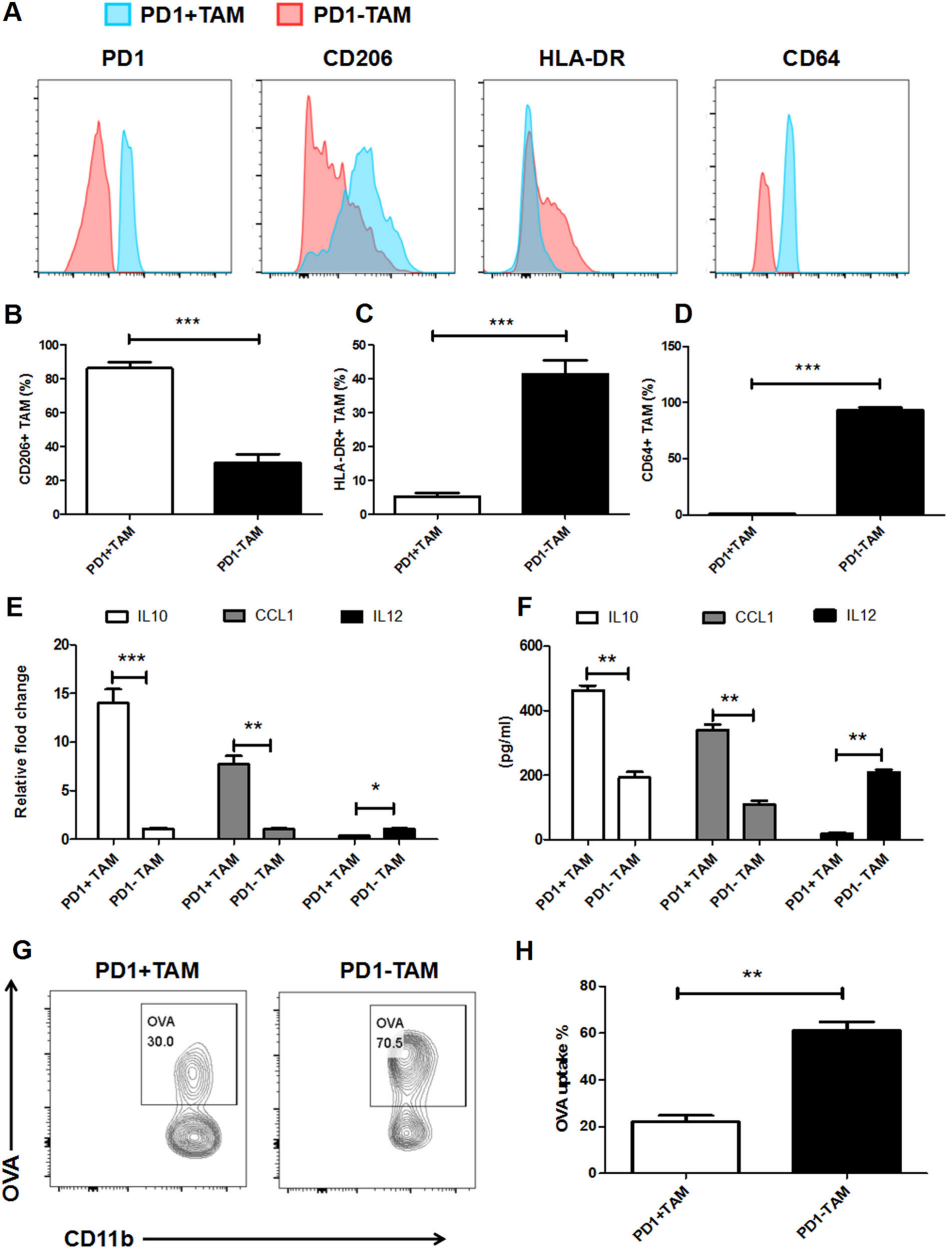
Phenotypic and functional characteristics of PD1^+^ macrophages in GC tissue. **a**–**d** Flow cytometry analysis was performed to analyze the phenotypic characteristics of PD1^−^, and PD1^+^ macrophages from GC tissues. **a** Analysis of representative markers expressed by tumor-infiltrating PD1^−^, and PD1^+^ macrophages. **b**, **c** Results represent the mean of four independent experiments. **e**, **f** Analysis of IL-10, IL-12, and CCL1 expression in PD1^−^, and PD1^+^ macrophages derived from GC tissue by q-PCR (**e**) and ELISA (**f**). **g**, **h** Phagocytic ability analysis of PD1^−^, and PD1^+^ macrophages derived from GC tissue by flow cytometry. Representative flow cytometry plots are shown in (**g**). **h** Results represent the mean of five independent experiments. *P < 0.05, **P < 0.01, and ***P < 0.001 (Student's *t* test)

We compared the phenotypic features of GC-infiltrating PD1^+^ macrophages and PD1^−^ macrophages. FACS analysis of typical M1 and M2 markers confirmed that PD1^+^ and PD1^−^ macrophages from GC tissues expressed more of the M2-associated surface molecules such as CD206, and less CD64 and MHC class II (Fig. 2a–d).

To further determine the functional characteristics of PD1^+^ macrophages, we analyzed their cytokine expression using ELISA and q-PCR. Correspondingly, the PD1^+^ macrophages derived from GC tissues expressed higher levels of M2 markers IL-10 and CCL1, but not M1 marker IL-12p70, than PD1^−^ macrophages (Fig. 2e, f). We then evaluated the phagocytosis levels of PD1^+^ macrophages by analyzing the proportion of OVA^+^ macrophages. FACS analysis of PD1^−^ vs. PD1^+^ macrophages showed that PD1^+^ macrophages phagocytosed significantly lower OVA in vitro than PD1^−^ macrophages (Fig. 2g, h).

### PD1^+^ macrophages exhibit immunosuppressive activity and triggering PD1 enhances this suppressive function

**Fig. 3:**
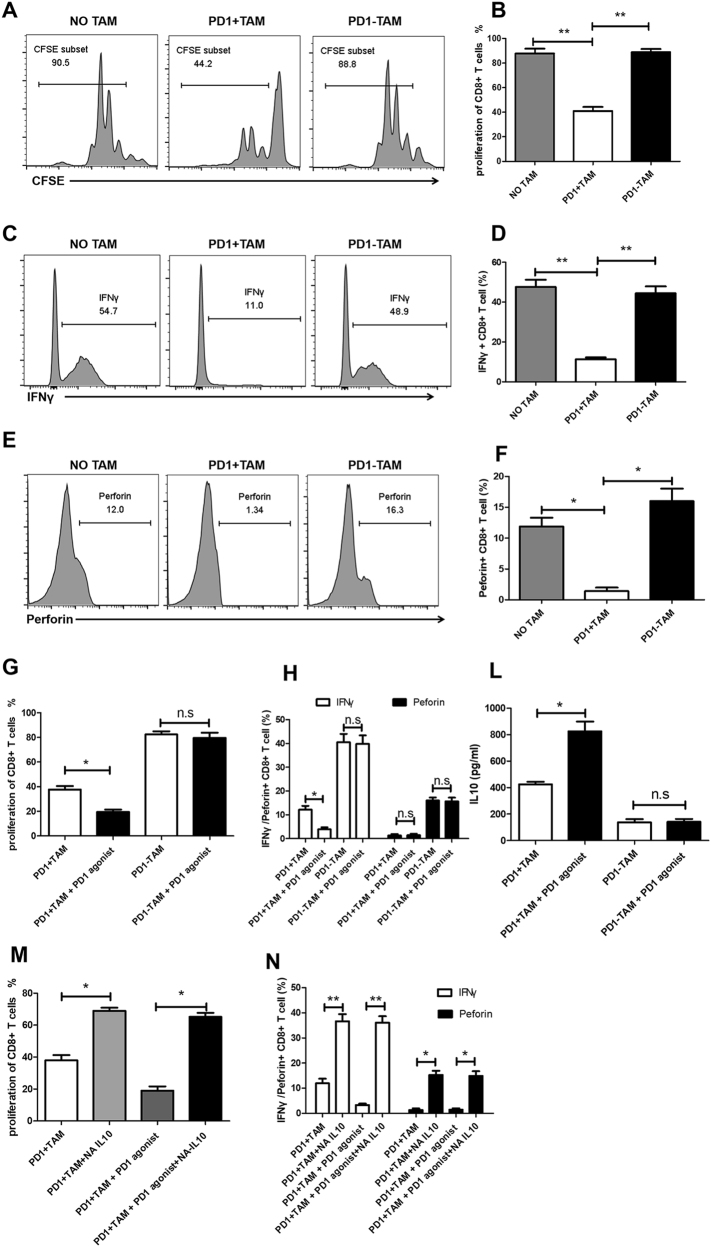
PD1^+^ macrophages exhibit strong immunosuppressive activity. **a**, **b** CFSE-labeled CD8^+^ T cells were activated by anti-CD3/CD28 beads, and PD1^−^, and PD1^+^ macrophages derived from GC tissue were added at 1:1 ratio. After 3 days, these cells were stained with anti-CD8 Ab, and the proliferation of CD8^+^ T cells in NO TAM (CD8^+^ T cells were cultured alone), PD1^+^ TAM (CD8^+^ T cells were cocultured with PD1^+^ macrophages), and PD1^−^ TAM (CD8^+^ T cells were cocultured with PD1^–^ macrophages) was analyzed. Representative flow cytometry plots are shown in (**a**). **b** Results represent the mean of three independent experiments. **c**, **d** Isolated CD8^+^T cells were activated by anti-CD3/CD28 beads, and PD1^–^, and PD1^+^ macrophages derived from GC tissue were added at 1:1 ratio. After 3 days, these cells were labeled with anti-CD8 and IFN-γAbs, and the expression of IFN-γ in CD8^+^ T cells in NO TAM, PD1^+^ TAM, and PD1^–^ TAM was analyzed. Representative flow cytometry plots are shown in **c**. **d** Results represent the mean of three independent experiments. **e**, **f** Isolated CD8^+^T cells were activated by anti-CD3/CD28 beads, and PD1^–^, and PD1^+^ macrophages derived from GC tissue were added at 1:1 ratio. After 3 days, these cells were collected and stained with anti-CD8 and perforin antibody, and the expression of perforin in CD8^+^ T cells in NO TAM, PD1^+^ TAM, and PD1^–^ TAM was analyzed. Representative flow cytometry plots are shown in (**e**). **f** Results represent the mean of three independent experiments. **g, l** Analysis of suppressive activity by PD1^+^ macrophages left untreated or incubated with 10 μg/ml anti-PD1 antibody, or polyclonal goat IgG. **g** CD8^+^T-cell proliferation was analyzed, and the data indicate the mean ±  standard error of the mean of three independent experiments. **h** The expression of Perforin and IFNγ in CD8^+^T cells was analyzed, and the data indicate the mean  ± standard error of the mean of three independent experiments. **l** the expression of IL10 in PD1^+^ macrophages was analyzed, and the data indicate the mean ± standard error of the mean of three independent experiments. **m,n** analysis of suppressive activity by PD1^+^ macrophages treated with NA-IL10 in the presence of 10 μg/ml anti-PD1antibody, or polyclonal goat IgG. **m** CD8^+^T-cell proliferation was analyzed, and the data indicate the mean ± standard error of the mean of three independent experiments. **n** the expression of Perforin and IFNγ inCD8^+^T cells was analyzed, and the data indicate the mean ± standard error of the mean of three independent experiments

To investigate whether PD1^+^ macrophages could inhibit the function of CD8 T cells, human autologous peripheral blood CD8^+^ T cells were activated by anti-CD3/CD28 mAb beads, and PD1^+^ macrophages and PD1^−^ macrophages were added at 1:1 ratio. The result showed that PD1^+^ macrophages significantly reduced the proliferation of CD8 T cells (Fig. 3a, b). Moreover, IFN-γ and perforin expression in CD8^+^ T cells decreased distinctly in the PD1^+^ macrophage group compared with the PD1^−^ macrophage group (Fig. 3c–f).

We also analyzed whether the PD1 signal contributes to the suppressive effects of PD1^+^ macrophages. A PD1^−^-specific agonist significantly enhanced the ability of the PD1^+^ macrophages to inhibit the proliferation (Fig. 3g), and IFN-γ and perforin expressions of CD8^+^ T cells (Fig. 3h). These findings reveal a novel mechanism involving PD1^+^ macrophage suppressive function mediated by the PD1 signal.

### PD1^+^ macrophages are negatively correlated with CD8 T cells in GC tissue

**Fig. 4:**
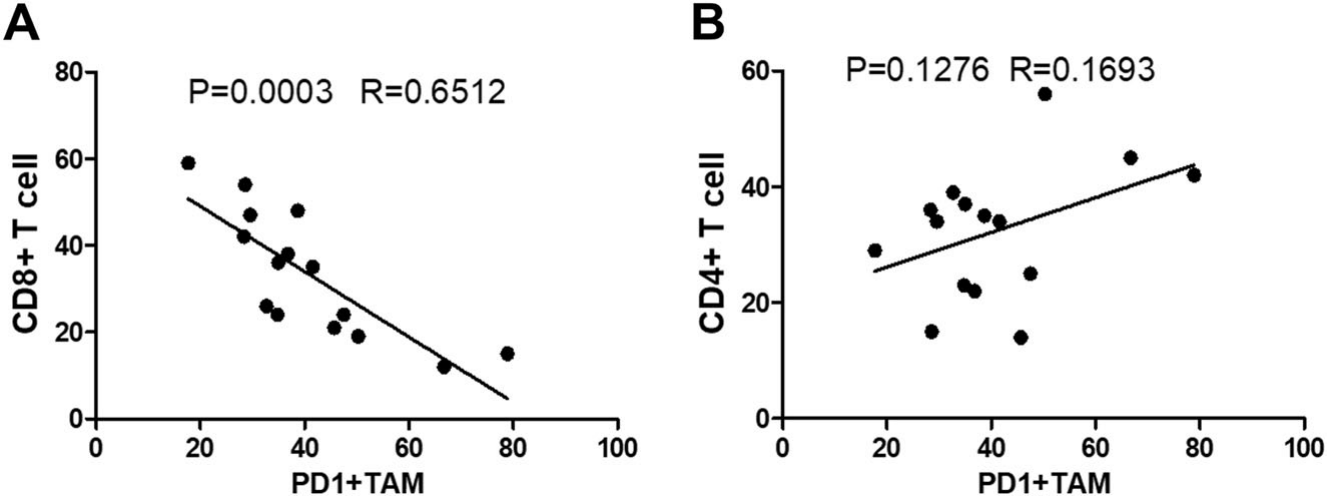
PD1^+^ macrophages are negatively correlated with CD8 T cells in GC tissue. **a**, **b** Flow cytometry analysis of PD1 expression on macrophages and the ratio of CD4^+^ or CD8^+^ T cells in CD45^+^ immune cells from the GC tissue samples. **a**, **b** Quantification indicating the associations between PD1^+^ macrophages and CD4^+^ or CD8^+^ T cells in GC tissue

To analyze the potential function of PD1^+^ macrophages in immune cells, we investigate the correlation between the percent of PD1^+^ macrophages and lymphocytes in GC tissues. The results showed that the percent of PD1^+^ macrophages was negatively correlated with CD8^+^ T cells (Fig. 4b), but was not correlated with CD4^+^ T cells (Fig. 4a). These data showed that the protumorigenic activity of PD1^+^ macrophages might be mediated via influencing CD8^+^ effector T cells.

### Tumor-derived exosomes can promote PD1^+^ macrophages expansion from monocytes in vitro and in vivo

Interestingly, recent studies demonstrated exosomes, in particular, tumor-cell-derived exosomes, a plethora of immunomodulatory activities on dampening T-cell responses. Thus, we hypothesized that gastric-cancer cells-derived exosomes can induce PD1^+^ macrophage production.

**Fig. 5:**
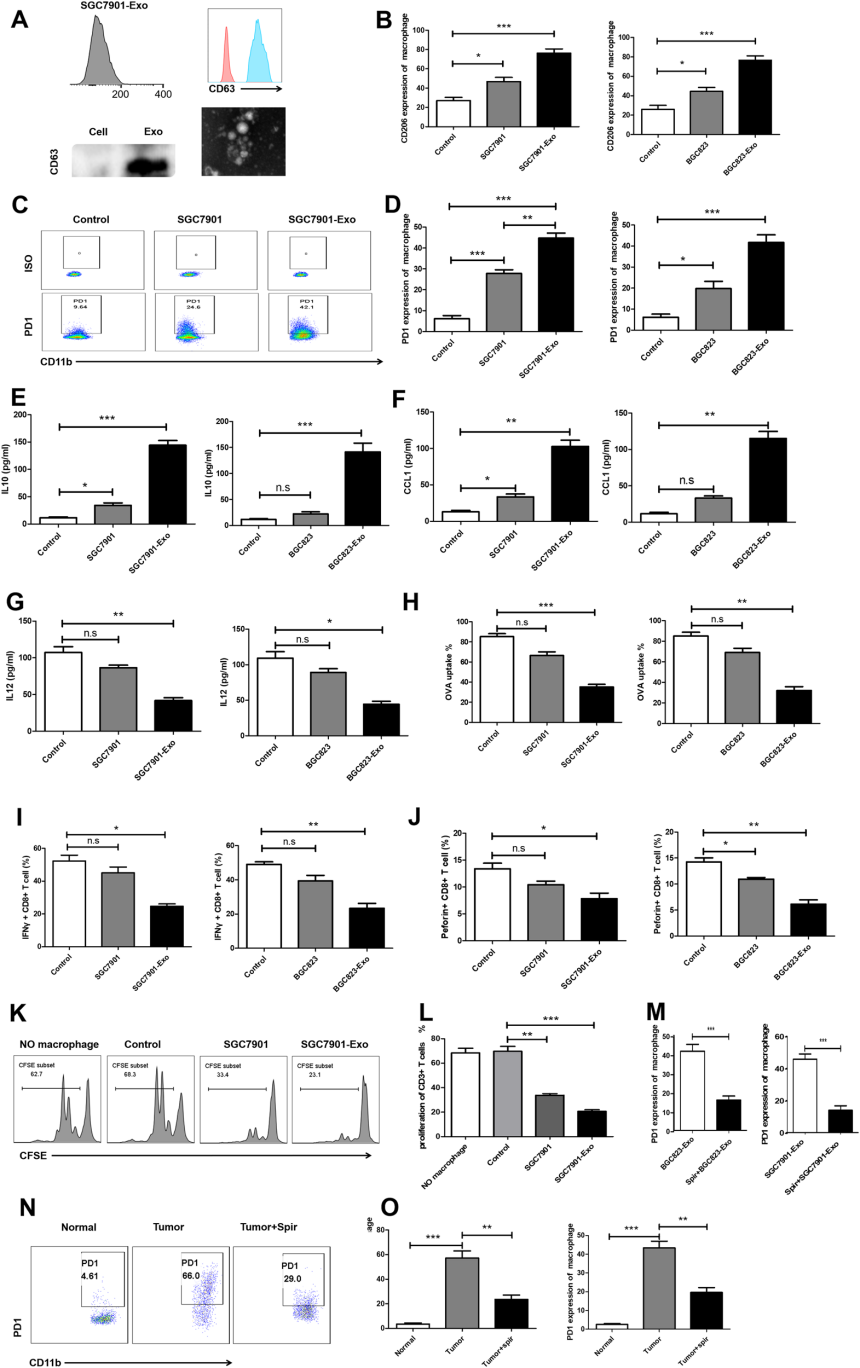
Tumor-derived exosomes promote PD1^+^ macrophages expansion from monocytes in vitro and in vivo. **a** Isolated exosomes were characterized by Nano-sight, western blot, and flow cytometry. **b**–**d** On day 3, monocytes cultured with or without exosomes were evaluated by flow cytometry to assess the frequency of PD1, and CD206 in monocytes. Representative FACS plots are shown in (**c**). **b**, **d** Results represent the mean of three independent experiments. **e**–**g** Analysis of IL-10 (**e**), IL-12p70 (**g**), and CCL1 (**f**) expression in monocytes cultured with or without exosomes by ELISA. Results represent the mean of three independent experiments. **h** Phagocytic ability analysis of monocytes cultured with or without exosomes by flow cytometry. Results represent the mean of three independent experiments. **i**–**l** Suppressive ability analysis of monocytes cultured with or without exosomes by flow cytometry. Results represent the mean of three independent experiments. **m** On day 3, monocytes cultured in the presence of exosomes that were collected from SGC7901 with or without the exosome release inhibitor spiroepoxide were evaluated by flow cytometry to assess the frequency of PD1. **n**, **o** FACS analysis of PD1 expression on macrophages from the samples as follows: non-tumoral tissue and tumor tissue from tumor-bearing mouse. Representative FACS plots of macrophage PD1 expression are shown in (**n**). Frequencies of PD1^+^ macrophages relative to total macrophage frequencies are shown in (**o**)

To evaluate the role of GC-derived exosomes in promoting PD1^+^ macrophage expansion, we exposed monocytes to secreted exosomes isolated from SGC7901 gastric cancer cell line. The isolation of exosomes was confirmed by Nano-sight, western blotting, and flow cytometry for the exosomal marker CD63 (Fig. 5a). Treatment with exosomes resulted in an increase in PD1 expression of monocytes (Fig. 5c, d). Moreover, exosomes coculture also led to the marked upregulation of the M2-associated scavenger receptor CD206 (Fig. 5b).

To further confirm the role of exosomes in the production of PD1^+^ macrophages, we determined their cytokine expression patterns, phagocytosis levels, and immunosuppressive activity. Exposure of monocytes to exosomes from SGC7901 and BGC823 led to an increase in IL-10 (Fig. 5e) and CCL1 expression (Fig. 5f), but a decrease in IL-12p70 expression (Fig. 5g). Moreover, FACS analysis showed that exosome-treated monocytes phagocytosed significantly lower OVA than monocytes cultured alone (Fig. 5h). The downregulation of IFN-γ, and perforin expression (Fig. 5i, j) and an increased ability to suppress CD8^+^ T-cell functions was also observed in exosome-treated monocytes (Fig. 5k, l). Importantly, treatment of monocytes and SGC7901 co-cultures with the exosome release inhibitor spiroepoxide led to a distinct reduction in the proportions of PD1^+^ macrophages following exposure to SGC7901 (Fig. 5m). We used a tumor-bearing mouse model to analyze the induction of PD1^+^ macrophages by tumor exosomes, and found that the tumor could induce PD1^+^ macrophage generation and that the exosome release inhibitor spiroepoxide could impair these effects (Fig. 5n, o). Thus, these results show that GC efficiently induces PD1^+^ macrophages from circulating monocytes by the secretion of exosomes.

## Discussion

Here, we reported an unrecognized immunosuppressive PD1^+^ macrophage subset and depict the phenotype, biological function, mechanisms of induction, and clinical relevance of those cells in GC patients.

The PD1 signal is one of the best-studied and most clinically successful immune-checkpoint drug targets, and its primary function is widely understood to regulate T-cell function. However, the expression and role of PD1 on other immune cells is poorly understood. Previous studies have reported that macrophages expressed significantly higher levels of PD1 during the context of pathogen infection^12–14^, which resulted in macrophage dysfunction. Here, we provide evidence that TAMs from GC patients also share markedly increased PD1 levels, which promotes GC progression by impairing the antitumor functions of CD8^+^ T cells. These data are consistent with the observation that TAMs from colorectal cancer increase PD1 expression and that PD1 expression inhibits TAM′s phagocytosis and tumor immunity^15^.

TAMs have been found to polarize toward an inflammatory M1 or pro-tumor M2 state, depending on their environmental stimuli^26^, and distinct TAM populations differed at the surface profile and function^27^. Our results show that PD1^+^ TAMs express an M2-like surface molecule, such as a significant increase in the CD206, IL-10, and CCL1 expression, and a clear decrease in the MHC class II, CD64, and IL-12 expression and the ability to phagocytose OVA protein, whereas PD1^−^ TAMs show a trend toward the expression of an M1-like surface molecule. Functionally, PD1^+^ TAMs possess stronger immunosuppressive activity of CD8^+^T-cell function compared with PD1^−^ TAMs, and this suppressive function can be effectively enhanced upon PD1 triggering. These results suggest that PD1 signal immunotherapies may also function through a direct effect on PD1^+^ TAMs, with substantial implications for the treatment of GC patients with these agents.

We try to understand the underlying mechanism contributing to the accumulation and activation of PD1^+^ TAMs. With the emergence of exosomes as central players in tumor progression and metastatic niche formation and their expanding list of reported immune-regulatory activities^28–33^, we hypothesized that exosomes derived from tumor could play a key role in regulating macrophage activation during GC progression. Interestingly, treatment of monocytes with exosomes derived from gastric-cancer cells led to an increase in the PD1 expression on monocytes. A significant increase in the CD206, IL-10, and CCL1 expression, and a clear decrease in the MHC class II, CD64, and IL-12 expression were observed in exosome-treated monocytes. Moreover, functional analysis shows that an increased capacity to suppress autologous CD8^+^T-cell proliferation and IFN-γ expression was observed in exosome-treated monocytes. Moreover, the exosome release inhibitor spiroepoxide led to a distinct reduction in the proportion of PD1^+^ macrophages following exposure to exosomes. We used a tumor-bearing mouse model to analyze the induction of PD1^+^ macrophages by tumor exosomes, and found that the tumor could induce PD1^+^ macrophage generation and that the exosome release inhibitor spiroepoxide could impair these effects. These results show that gastric-cancer cells dramatically educate monocytes into PD1^+^macrophages through the secretion of exosomes in vitro and in vivo.

Collectively, our data provide a new insight into possible manipulation of PD1^+^ TAM-mediated immunosuppression in gastric cancer. Exosomes derived from gastric-cancer cells effectively induce the production of PD1^+^ TAMs. After interacting directly with PDL1^+^ cells, the PD1^+^ TAMs produce a large number of IL-10, induce the dysfunction of CD8^+^ T cell, and thereby create conditions that are favorable to GC progression. Accordingly, methods in which immunotherapy is combined with targeting PD1^+^ TAMs and tumor-derived exosomes should be used to restore immune function in GC patients.
