# Correction: Tumor-derived exosomes induce PD1^+^ macrophage population in human gastric cancer that promotes disease progression

**DOI:** 10.1038/s41389-022-00381-y

**Published:** 2022-02-08

**Authors:** Furong Wang, Bin Li, Yucai Wei, Yang Zhao, Li Wang, Peng Zhang, Jinwei Yang, Wenting He, Hao Chen, Zuoyi Jiao, Yumin Li

**Affiliations:** 1 Department of Pathology, Lanzhou University Second Hospital, Lanzhou University Second Clinical Medical College, Lanzhou, China; 2 Gansu Provincial Key Laboratory of Digestive System Tumors, Lanzhou University Second Hospital, Lanzhou University Second Clinical Medical College, Lanzhou, China; 3 School of Life Sciences, Lanzhou University, Lanzhou, 730000 China; 4 Department of Thoracic Surgery, Lanzhou University Second Hospital, Lanzhou University Second Clinical Medical College, Lanzhou, China; 5 School of Basic Medical Science, Lanzhou University, Lanzhou, 730000 China; 6 Department of General Surgery, Lanzhou University Second Hospital, Lanzhou University Second Clinical Medical College, Lanzhou, China

Correction to: *Oncogenesis* 10.1038/s41389-018-0049-3, published online 25 May 2018

Following the publication of this article, it was noted that incorrect images were inadvertently used for the merged figure in 1d and to represent SGC7901-Exo in 5c. The correct images have now been provided and the figures corrected.

The authors confirm these errors have no effect on the conclusions of the article.

**Fig. 1:**
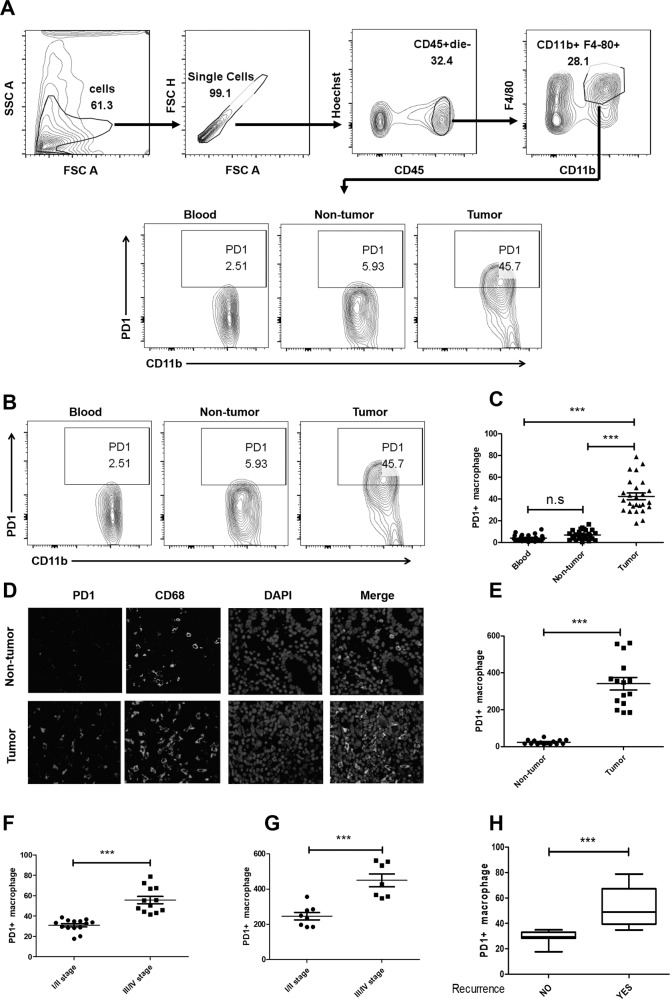
▉.

**Fig. 5:**
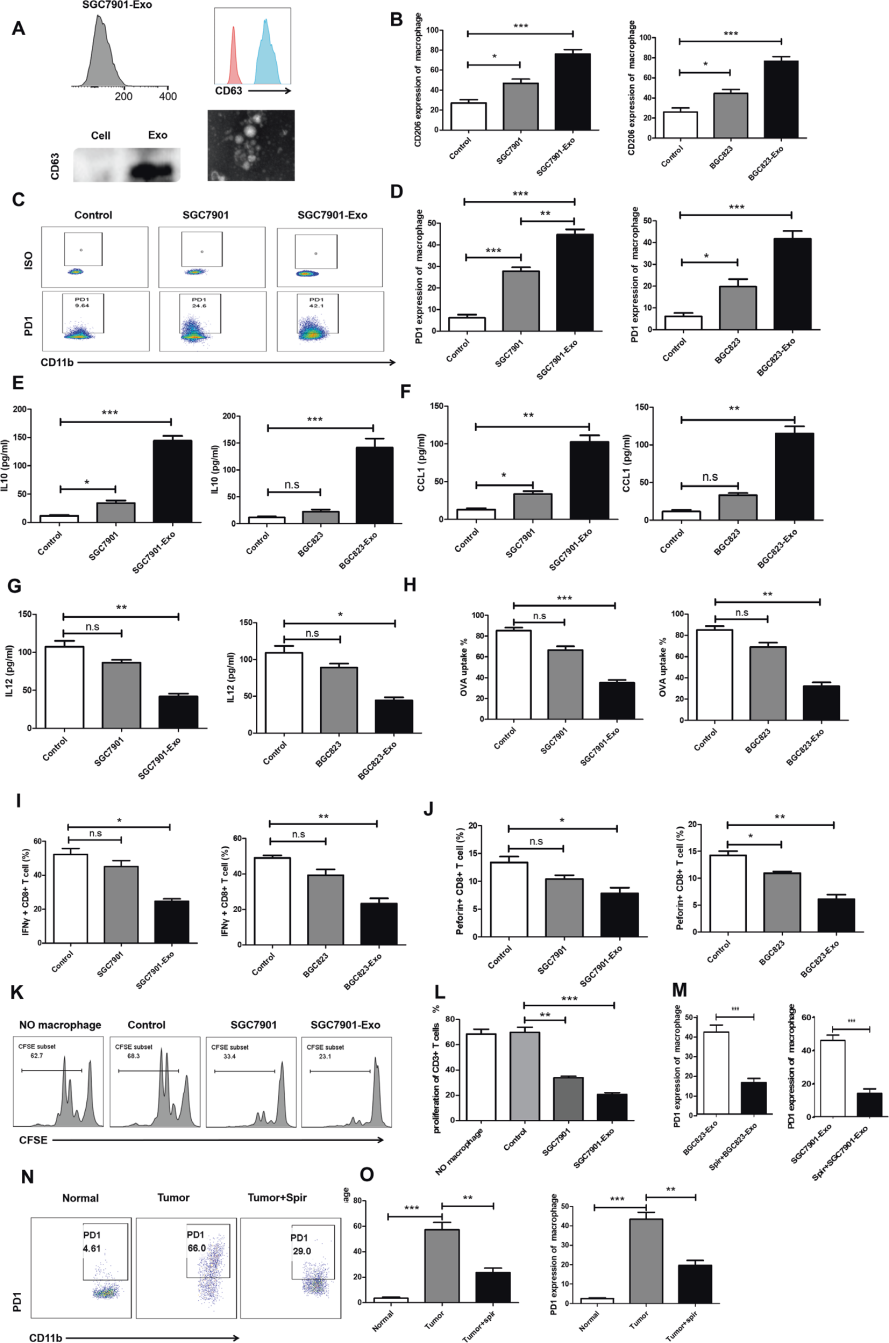
▉.

